# Urétérohydronéphrose géante: une cause exceptionnelle d’occlusion digestive

**DOI:** 10.11604/pamj.2013.15.61.2814

**Published:** 2013-06-20

**Authors:** Issam Yazough, Karim Ibn majdoub Hassani

**Affiliations:** 1 Faculté de médecine et de pharmacie de Fès, Université Sidi Mohammed Ben Abdellah, département de Chirurgie, CHU Hassan II Fès, Maroc

**Keywords:** Hydronéphrose, jonction pyélo-urétérale, rein, occlusion digestive, néphrectomie, hydronephrosis, ureteropelvic junction, kidney, intestinal occlusion, nephrectomy

## Image en médecine

L’hydronéphrose est une dilatation du bassinet le plus souvent par syndrome de la jonction pyélo-urétérale. Elle peut se compliquer de calcul, d’infection ou de destruction rénale. Son traitement est chirurgical. Nous présentons le cas d’un patient âgé de 47 ans, sans antécédents pathologiques notables admis aux urgences dans un tableau d’occlusion intestinale aigue fait d’arrêt des matières et des gaz depuis trois jours avant son admission. L’examen clinique objective un abdomen très distendu avec une matité à la percussion et une ampoule rectale vide au touché rectal. La radiographie d’abdomen sans préparation a objectivé quelques niveaux hydro-aériques latéralisés à droite. Le bilan biologique était sans particularité en dehors d’une CRP à 225. La fonction rénale était normale. La TDM abdominale montre une énorme formation liquidienne multi cloisonnée qui occupe toute la région abdomino-pelvienne latéralisé sur le coté gauche refoulant les structures digestives vers le coté droit et témoignant d’une importante dilatation des cavités excrétrices du rein gauche avec absence de visualisation de ce dernier. La TDM montre par ailleurs une hypertrophie compensatrice du rein droit. Une néphrostomie a été réalisée en urgence puis le malade a bénéficié d’une néphrectomie quelques jours plu tard.

**Figure 1:**
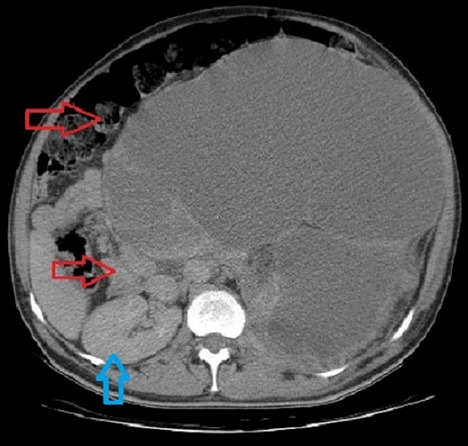
Urétérohydronéphrose géante refoulant toutes les structures digestives à droite (flèche rouge), légère hypertrophie compensatrice du rein droit (flèche bleu)

